# Understanding the public temper through an evaluation of rumours: an ethnographical method using educational technology

**DOI:** 10.1057/s41599-018-0197-2

**Published:** 2018-11-27

**Authors:** Abdulrahman Essa Al Lily, Shaher R. Elayyan, Ahmed Ali Alhazmi, Saleh Alzahrani

**Affiliations:** 1 Department of Educational Technologies, King Faisal University, Saudi Arabia, Post Box: 346, Al Ahsa, 31982 Saudi Arabia; 2 Department of Curricula and Instruction, King Faisal University, Saudi Arabia, Post Box: 400, Al Ahsa, 31982 Saudi Arabia; 3 Department of Education, Jazan University, Saudi Arabia, 6487 Ash Shati, Jazan, 82912-2822 Saudi Arabia

**Keywords:** Sociology, Social anthropology

## Abstract

The power of rumours is that they can be broadly exchanged, generating a ‘public temper’ (which is everybody’s temper without being anybody’s temper in particular). This article, therefore, describes an approach to measuring the public temper, examining particularly the public temper of an Arab society, namely Saudi Arabia. It addresses the following research question: is it possible to analyse existing (scholarly) rumours to see if they can be used as informants of the public temper of the culture in which they exist? This question is answered ethnographically by analysing 579 Arabic online rumours collected by students as part of their critical engagement with educational technology. Having analysed the data, four categories emerged: the *concerns*, *interests*, *attitudes* and *values* of Saudi Arabia. According to the literature, these four categories, taken together, constitute the *emotional* domain (i.e., the public temper) of a society. Thus, a theoretical proposition (and contribution to the existing literature regarding sociology) is that rumours mirror the public temper of a culture, reflecting a range of emotions from simple to complex (from concerns, interests and attitudes to values). Simpler emotions (e.g., concerns) appear to be more easily affected by rumours than more complex emotions (e.g., values). An implication of this study is that rumours have ‘biographies’, which detail public tempers across space and time. Rumours are ‘records’ of public tempers that should be read in the same way archaeologists read landscapes and remains. Although rumours entail *ill-defined* information, it is feasible to *well define* society through such ill-defined information, meaning that something can come out of its opposite. This study offers ethnographers a new method of understanding public tempers through rumours, alongside conventional meaning-making symbols (e.g., poems).

## Introduction

Rumours can be widely exchanged, thus producing a ‘public temper’, which is ‘everybody’s without being anybody’s in particular’ (Durkheim, 1947: 102). This article, therefore, describes an approach to measuring public tempers. Social media are a fertile ground for the dissemination of rumours (Tufekci, 2008). Through social media, rumours have experienced a high level of diffusion and have become more influential than in the past, thereby becoming worthy of serious academic research and scholarly attention (Mintz, 2002). Rumours can sometimes be limited and weak but, at other times, they can hit hard, initiating battles and bringing down political systems (cf. Caplow, 1947; Lotan et al. 2011). The effect of rumours can be destructive (Fine and Heath, 2009), and yet it arguably takes time for this destruction to unfold. A concern is that, if a rumour changes public opinion, and then the rumour is discredited, the changed public opinion can remain the same, despite being based initially on false information. Rumours stick easily in the mind and hence people will mentally and subconsciously go back to them. Put simply, ‘Once we’ve heard an untrue claim, we can’t simply unhear it’ (Harford, *Financial Times*, 9 March 2017). Echoing these arguments, this article examines how rumours can help us understand the public temper of a society. It first establishes a conceptual framework for the study, explaining the key existing terms and concepts concerning rumours and presenting the existing kinds of writing on rumours. It then talks about the methodological aspects of this study, setting out the data collection and analysis methods. After that, it presents the key findings and engages in interpretation of these findings. It then discusses these findings and links them back to the literature. Finally, it offers concluding remarks, which outline the research in plain language, show the limitations and suggest further research paths.

## Conceptual framework

The term ‘rumour’ refers to ‘information or a story that is passed from one person to another and which *may* or *may not* be true’ (*Longman Dictionary of Contemporary English*, emphasis added). Rumours may be ‘the oldest medium of mass communication of information or ideas’ (Kapferer, 1990: 1). The literature addresses rumours in relation to three types of information: misinformation, disinformation and, indeed, accurate information (Rosnow and Fine, 1976; Lee, 2014). While misinformation refers to *unintentionally* made false information, disinformation signifies *deliberately* made false information. In other words, misinformation comes out of limitations in human communication, while disinformation comes out of the human predilection for shaping one’s surroundings in one’s own interests. Disinformation, unlike misinformation, has the subjective intention to deceive and direct (Jowett and O’Donnell, 2006). It is the outcome of deliberate lies that seek to mislead. Misinformation is the result of honest mistakes. However, the current research is not interested in unearthing the reasons behind inaccurate information. Rather, it looks into what rumours tell us about the nature of the society where they have existed.

Studies have looked into the linguistic aspects of rumour and have outlined a number of terms that people tend to confuse with the term ‘rumour’. One such term is ‘humour’. Humour is intended to make one laugh, but rumour is meant to make one believe (Knapp, 1944; Allport and Lepkin, 1945). Another confusing term is ‘gossip’. Gossip is concerned with private, trivial, pieces of information, whereas rumour is associated with public matters (DiFonzo and Bordia, 2007). That said, there is overlap between these two terms and this overlap is increased by such modern ideas as ‘infotainment’ and ‘tabloidisation’. Infotainment refers to information that aims to both inform and entertain (Thussu, 2008). In tabloidisation, journalism does not focus only on politics and foreign affairs but also on entertainment and celebrities (Turner, 1999). Considering these linguistic clarifications, the current study will look into rumour (not humour); into what seeks to be believed (not what provokes laughter). It concentrates more on rumours (not gossip) that relate to public, social matters (not the private and personal).

There are certain terms associated with rumours. One is ‘propaganda’, which is subjective information intended to trigger emotional (instead of rational) responses so as to encourage particular syntheses, influence audiences, further agendas, promote political causes and alter attitudes towards specific positions (Ross, 2002). Another term is ‘spin’, which is a type of propaganda associated with the psychological mechanisms of using exceedingly deceitful, misleading and controlling strategies, providing highly biased interpretations of events and/or campaigning to persuade public opinion in accord with or opposition to certain organisations or public figures (Branigan, *The Guardian*, 12 June 2007). An additional term is ‘fallacy’ or ‘fallacious reasoning’, which is the application of deficient, untrue or flawed reasoning when building up arguments (Bustamente and Dahlman, 2015). ‘Circular reporting’ refers to a condition wherein a piece of information seems to originate from multiple independent sources despite actually originating from one single source. ‘Doublespeak’ is a term that means intentionally reversing, masking, covering up and twisting the meaning of terms (Kehl and Livingston, 1999). ‘Hoaxes’ are deliberately fabricated false statements made to masquerade as the truth (MacDougall, 1958). ‘Fabrication’ is used in scholarly enquiry to refer to the deliberate misrepresentation of research findings by making up data. ‘Gaslighting’ is a type of manipulation that attempts to spread self-doubt in individuals or communities, with the intention of making them rethink their own memory, insight and understanding (Dorpat, 1994). This article is about rumours, not ‘contemporary legends’, which have been written about by sociologists, such as Fine (1980) and Best (1997), and indeed by folklorists, historians and psychologists.

There are three types of research into rumours. Type 1 is *politically* oriented research, looking into political issues surrounding rumours and into the political use of rumours (Briant, 2015). For example, Coast and Fox (2015) have shown the key role of rumours in politics, proving that the act of spreading negative rumours about rivals tends to be more successful than the act of promoting positive messages about one’s own party. Type 2 is *psychologically* driven research, which studies cognitive matters related to rumours. By way of illustration, Bordia and DiFonzo (2004) have argued that rumour transmission is reflective of a collective explanation process and that rumours are ‘sense-making’ statements and attempts at solving problems. Type 3 is a *lawfully* oriented investigation, examining legal topics in relation to rumours. For instance, Sunstein (2009) has discussed rumours in line with their risk to personal reputations and according to libel and privacy laws. What is missing among these approaches, however, is a *culturally* driven type that examines the cultural dimension of rumours. The present research aims to fill this gap, by looking into the relationship between rumours and the culture wherein they exist. Previous studies have focused on the meanings and uses of rumours, whereas the current study looks into the *implications* of rumours, at what rumours culturally *imply*. Hence, the research examines whether it is possible to analyse rumours to see if they can be informants of the culture in which they exist.

## Methodology

The current study addresses the following research question: Is it possible to analyse existing (scholarly) rumours to see if they can be used as informants of the public temper of the culture in which they exist? It concentrates on what could be called ‘scholarly rumours’, which are defined in the current article in two ways. First, they are rumours that present causality, in that they show relationships between causes and effects. Second, they are rumours falsely based on research and experimentation. Scholarly rumours are, arguably, influential, considering that society tends to trust scholarliness. This social trust remains because scholarliness has long been an agent for change, having the power to initiate changes in wider society and push or break down cultural boundaries (Corbyn, *Times Higher Education*, 5 November 2009). Besides, scholarliness is associated with expertise and has been proved to contribute to the ‘progress’ of human society and the development of standards of living. Another reason for concentrating on scholarly rumours is that they, unlike non-scholarly rumours, have a tendency to sound more ‘sophisticated’, to last for a longer time, and even be transmitted from one generation to another.

Scholarly rumours do not come from nowhere, nor are they without political intentions (Lasswell, 1938). They do not happen in a vacuum but are influenced by social norms, cultural values and political agendas. This implies that scholarly rumours can be reflections and, moreover, reinforcers of social realities. The norms and values of a culture can be drivers and subjects of scholarly rumours. Scholarliness can, hence, be misused as a tool in daily social and political games. The scholarly language can be spoken to gain social sympathy. A concern is that, when scholarly rumours are well incorporated into the surroundings, they then become ‘naturalised’ and hidden in the landscape (Bernardi et al. 2012), resulting in neglect of the politics exercised by them. Scholarly rumours can be implicitly objectified in, and ‘wrapped’ into, social media, penetrating the social fabric and moving across generations. If scholarly rumours are documented and written down, they act as archives and therefore spaces in which certain kinds of power are codified and justified. With these observations in mind, it would be politically naïve to attempt to conceptualise scholarly rumours in isolation from the political intentions behind them. To interpret and discuss scholarly rumours as isolated elements is to risk treating them as being outside the social fabric. It is therefore well worth considering both the ‘culturalisation’ of scholarly rumours and the ‘rumourisation’ of cultures.

The research question is addressed within an Arab context (Saudi Arabia) that has seen the founding of ‘anti-rumour’ associations, campaigns, hashtags, workshops and studies (Al Qarnaf, 2014; Al Lily et al. 2017). Many members of Saudi Arabian society enjoy ‘journalistic curiosity’, i.e., they care about knowing what others do. Saudi Arabian society is a collective and communicative community where everyone cares about everyone, which makes it easier for rumours to spread. In other words, rumours are used in Saudi Arabian society to aid social bonding, solidarity and community (cf. Miller, 1992; Dunbar, 2004). Many Saudi Arabs seemingly have both a desire to communicate and an unwillingness to read. So, when they encounter lengthy messages, they combine their desire to communicate with their unwillingness to read by forwarding these messages without reading them deeply or checking their accuracy.

For some Saudi Arabs, the *act* of passing around information (regardless of its trustworthiness and credibility) is a cultural virtue, making the distribution of rumours a socially well-received act. Many Saudi Arabs forward any information (regardless of accuracy) that displays *warnings* against something, because of their over-concern and over-care about family and friends. Spreading rumours is seen as a means of enhancing communication among family members and friends, given that such communication has social and cultural value (and is even an obligation). Many Saudi Arabs may not realise the implications and ramifications of rumours, and therefore they exchange and spread them merely for fun and to kill time, socialise and overcome boredom. Many Saudi Arabs like rumours because they see them as ‘fast food’—i.e., like easy-to-reach and consume ‘ready meals’. Because information is censored and, therefore, limited, it is less available and this, therefore, encourages rumours. Moreover, the relationship between Saudi individuals and managers is not harmonious, and hence some individuals use rumours as an indirect tool to criticise or challenge managers. In Saudi Arabian organisations, transparency tends to be limited, encouraging members to come up with rumours through which they place pressure on managers to reveal truths and be more transparent. Studying rumours in a collective society is different, and the concern is that some readers of this article (who come from an individualistic culture) may default to individualist modes of thinking.

This article seeks to study how male Saudi Arabian undergraduates perceive a broader trans-national Arabic-writing audience on the Internet. In 2017, five groups of 20 Saudi Arabian undergraduates (a total of 100 students) in a Saudi university participated in the study. Every effort was made to follow the best ethical practices, such as seeking to ensure that there was no harm to participants, no lack of informed consent, no invasion of privacy and no deception involved. Informed consent was obtained from candidates after explicitly describing the relevant features of the research and officially asking for their consent. The participants were then asked to collect rumours as part of their critical engagement with educational technology. They were social-science students, aged between 18 and 22 years, Saudi Arabian, male, single (except for two), undergraduates, unemployed (except seven part-time employees) and came from lower-middle-class or working-class families. There is, therefore, a bias towards the interests of Saudi Arabian undergraduates.

Saudi Arabian undergraduates were chosen for various *culturally specific* reasons. The researchers saw them as appropriate for the research because they were more exposed to the outside world than high-school teenagers and yet were less serious and conservative than graduates (Abdullah, 1986; Al Doori, 2008). For example, undergraduates tend to talk more freely about gossip, compared with Saudi graduates, who perceive their academic degree as making them ‘culturally responsible’ and ‘social examples’. To illustrate, asking Saudi Arabian graduates for input into any culturally ‘unethical’ topic (here, ‘gossip’) would be regarded by many such graduates as ‘insulting’ and ‘offensive’, because they would think that the researchers were assuming they were tolerant of unethical behaviour. Asking for one’s input into any culturally unethical topic would be seen by graduates as pointless, given that this topic is unnegotiable and culturally ‘settled’ to be negative. Saudi Arabian undergraduates, on the other hand, would be less likely to take offence.

The students were all ‘Arabs’—i.e., ‘members of a Semitic people, originally from the Arabian Peninsula and neighbouring territories’ (*Oxford Dictionaries*). Arabic was the language of instruction, and the students were all native speakers of Arabic. They were asked to use mobile phones during class-time (1 h and 40 min) to collect rumours via social networks (as part of their online curricular activities). Once one student found a rumour, he said it aloud immediately so as to reserve this rumour for himself. That is, one could not reserve any rumour that one’s peers had already reserved. A point was won for every rumour reserved. It was easy for Group 1 to collect rumours (and therefore collect points). Group 2, however, found it difficult to find rumours that had not already been found and therefore reserved by their peers in Group 1. Group 3 faced even more difficulty since their peers in Groups 1 and 2 had reserved so many rumours. Groups 4 and 5 reported finding it ‘impossible’ (in their words) to find rumours that were not already found and reserved by their peers in Groups 1, 2 and 3. Members of Groups 4 and 5 were unable to reserve more than two rumours, and therefore were unable to win more than two points. This is when the authors decided to stop the process of having more groups and collecting more rumours, since this perceived ‘impossibility’ of collecting more rumours was seen as an indication of having managed to collect common rumours during that particular period.

The students managed to collect 579 rumours in total. A key limitation in this research is, of course, that some of the participating students perceived some pieces of information to be accurate and therefore did not collect them, even though these pieces were actually rumours. The task of checking the accuracy of rumours is beyond the scope of this research. This research collects merely what *appear* to be rumours and what are *socially perceived* as rumours; that is, what the students thought of as rumours. So, the current study is associated with what is thought to be a rumour and is not intended to examine the accuracy of rumours. The authors designed the study and created the methodology in line with the work of Goodwin and Goodwin (2016), who discuss in-class research. It should be clarified that the research was not *on* students but rather *through* students—i.e., employing students as ‘data-collectors’ (Al Lily, 2013). While it is debatable whether it is ethical to employ one’s students for one’s research (Denzin and Lincoln, 1994), this exercise was seen as part of the students’ practical learning about research. There are various advantages of in-class data collection by student teams, including familiarity with the wider culture, a budget-friendly workforce and accessibility to diverse resources.

This is a sociology paper, showing how male Saudi undergraduates define rumours. As a methodology paper, it describes an approach to measuring the public temper among a group during a 100-min class. The collected rumours were analysed to see what they could tell us about Saudi Arabian culture. These rumours were first coded. The code for each rumour is basically the keyword of the rumour. Similar codes were then grouped to generate sub-categories. After that, similar sub-categories were assembled to generate categories. These categories, collectively, detailed the subject of the research (i.e., a theoretical proposition). Table 1 displays the whole analytical process. This table shows merely a sample or ‘taste’ of the rumours, codes and sub-categories. It shows only a selection of codes, given that there are a large number of codes. Some codes can belong to more than one sub-category. To enhance the quality of this table, three Arab and three non-Arab academics were asked to critically challenge and problematise them. The main advantage of having *Arab* academics review this table is that they are familiar with the social context under investigation. A key benefit of having *non-Arab* academics involved as well is that they are ‘outsiders’ in relation to the researched context, and hence they are more likely not to take things for granted or be biased.

**Table 1:** Analysis of the raw data

Examples of the rumours (Data)→	How many students reported the rumour	Codes→	Sub-categories→	Categories→	Theory
If one eats chewing gum, it will be in one’s *stomach* for seven years	16	Stomach ache	*Medical concerns*	*Concerns*	Rumours in a society can tell us about the public temper (i.e., concerns, interests, attitudes and values) of the society
Drinking tea after having just eaten causes *osteoporosis*	35	Osteoporosis			
If a pregnant woman likes salt, the baby is a *boy*. If she likes sugar, the baby is a *girl*	22	Gender	*Social concerns*		
If your ear makes a noise, this means that someone is *gossiping* about you	35	Gossip			
The signals of mobile *phones* can cause cancer	43	Phone	*Technological concerns*		
*TV remotes* harm children and pregnant women	12	TV Remote			
Eating both eggs and bananas can result in *death*	16	Death	*Biological concerns*		
*Lemons* can kill cancer cells and are 10,000 times stronger than chemotherapy	12	Cell			
Hanging a picture of horses means children will not experience *nightmares*	9	Nightmares	*Mythical concerns*		
If you eat a fruit *kernel*, a tree grows in your belly	5	Kernel			
There will be a rare, strong typhoon hitting Saudi Arabia, Yemen and Oman	26	Typhoon	*Environmental concerns*		
There will be an *asteroid* that will soon collide with Earth	15	Asteroid			
Having white hair is a sign of having a *healthy* body	18	Well-being	*Medical interests*	*Interests*	
Drinking hot water or olive-oil leaves *heals* diabetes	20	Healing			
Having sex in the morning makes one *optimistic* for the rest of the day	32	Optimism	*Psychological interests*		
Drinking buttermilk before leaving the house brings *luck*	10	Luck			
Pizza is originally *Arabian*	36	Arabism	*Social interests*		
How the male mentality functions is: 70% love-making, 10% how to make love, 10% who to make love with and 10% when to make love	13	Love-making			
Elephants are *scared of* mice	30	Power	*Political interests*		
An eye-shaped amulet can *protect* against the evil eye	30	Protection			
An EKG [i.e., a device recording the electrical activity of the heart over a period of time] in an American hospital has drawn the word ‘Allah’	12	Miracle	*Mythical interests*		
Holding wood helps one to achieve one’s *wishes*	17	Wish			
*Parsley* tea helps with leg tumours	19	Parsley	*Natural interests*		
Eating *aubergine* can help overcome a headache	12	Aubergine			
*Males*, like *women*, have hymens	27	Equality	*Cultural attitudes*	*Attitudes*	
*Only men* can be colour-blind	14	Difference			
Wadi Al Duwasir [i.e., a town populated by *black*-skinned people] is inhabited by ghosts	16	Racism	*Political attitudes*		
Sudanese people are *the laziest people*	31	Stereotypes			
If a woman drinks too much tea, she suffers from a lack of *emotion*	11	Emotion	*Psychological attitudes*		
If a pregnant woman *desires* to eat something but does not eat it, her baby will be born with a melanocytic nevus	28	Desire			
Touching cats causes *baldness*	33	Baldness	*Bodily attitudes*		
The over-use of mobile phones causes *blindness*	24	Blindness			
*Women* are better than men at maths because of the many hormones women have	23	Women	*Social attitudes*		
Only *men* watch pornography	5	Men			
A study shows that intelligent people stay up at night, and that stupid people go to bed early	21	Intelligence	*Educational attitudes*		
If your handwriting is bad, this is a sign of being a genius, because your mind is busy with more important matters than such a trivial thing as handwriting	9	Writing			
A scholar decoded the sounds of trees in forests and found that these sounds are a repetition of the word ‘Allah’. He then converted to Islam	22	Allah	*Cultural values*	*Values*	
If one takes pictures of a *ghost*, the ghost will kill you	6	Spirit			
Hair and nails continue to grow after death, in one’s *grave*	24	Grave	*Mythical values*		
When hitting a black cat, one will be *possessed*	8	Possession			
*Lying* causes tumours in one’s tongue	37	Honesty	*Social values*		
Eating lettuce causes *infertility*	22	Infertility			
Beetroot juice makes old people *feel young* and lively	7	Youth	*Psychological values*		
Eating at night makes one *fat*	16	Slimness			
The length of the male *penis* is related to the length of the nose, foot and finger	7	Penis length	*Sexual values*		
Most Kuwaiti women are *shemale*	4	Shemale			
Chocolate and Pepsi contain *pig* oil	16	Pig	*Environmental values*		
‘Ketchup’ toothpaste is not made out of tomato but instead out of blood, *alcohol* and urine	30	Alcohol			

## Findings and interpretations

This research investigates whether the public temper of a community can be recognised through its rumours. Having analysed hundreds of Arabic online rumours, four categories emerged: the *concerns*, *interests*, *attitudes* and *values* of an Arab society. These categories are displayed in Table 1. According to the literature, these four categories, taken together, form the *emotional* nature (i.e., the public temper) of any society (Bloom, 1956; Krathwohl, 1964). Therefore, a theoretical proposition could be that rumours mirror the *emotional* sphere of any culture, reflecting a range of emotions from simple to complex (from concerns, interests and attitudes to values). Other researchers from non-psychological backgrounds might find non-emotional categories. For this reason, further research should be conducted by non-psychologists to re-analyse the raw data and the subject matter.

This first category shows that rumours do not arise in a vacuum, but rather they are a reflection of various **concerns** in a community (see Table 1). For example, the rumour that ‘*Eating Lay’s potato chips causes cancer’* can be seen as a sign of a medical concern with cancer in the community. In other words, people may have resorted to rumours because of the human inability to cure certain diseases (e.g., cancer). Likewise, the rumour that ‘*A higher level of forgetting is a sign of having a higher level of intelligence*’ indicates a psychological preoccupation with forgetting. Put in general words, rumours have been exploited here to ‘cheer up’ individuals’ psychological concerns over human limitations (here, forgetting). In this case, rumours have been used to improve self-confidence and/or make people feel good about themselves—that is, feel good about their congenital flaw of forgetting. In this case, rumours have been used to fill gaps in ‘human perfection’ and to overcome various aspects of human shortage (here, forgetting). There seem also to be environmental concerns, which can be seen in such rumours as ‘*NASA has announced that there will soon be storms that prevent sunlight from reaching Earth for six days’*. In addition, the social concern over gender can be noticed in various rumours, such as the rumour that ‘*If the stomach of a pregnant woman is up, the baby is a boy. If it is down, the baby is a girl’*. In this rumour, the use of ‘up’ to refer to males could be seen as a reflection of male superiority in an Arab society, whereas the use of ‘down’ to refer to females mirrors socially perceived female inferiority. Hence, men may help spread this rumour so as to promote their superiority.

This second category demonstrates that scholarly rumours do not come from nowhere but can hold intentions and **interests** (see Table 1). For example, a political interest that sought to discredit a certain product can be seen in the rumour that ‘*Indomie* [an instant noodle brand] *results in brain damage*’. When one has a strong interest in a particular concept (such as the health risk of sugar and chocolate), one may feel excited about spreading any information (regardless of its accuracy) about this concept, such as the information that ‘*Mars bars contain pieces of plastic’*. At times, a rumour shows a positive social interest in something (e.g., music), whereas another rumour exhibits a negative social interest in this same thing. For instance, a rumoured positive interest in music is ‘*Cows produce more milk when listening to music’*, and a rumoured negative interest in music is ‘*If one listens to music, a skewer made of fire will be stuck into one’s ear’*. Sometimes, a rumour promotes a natural interest in something (e.g., dates), and yet another rumour enhances a natural *dis*interest in the same thing. For example, a promoted natural interest in dates can be seen in the rumour that ‘*Dates help to remove air gases from the womb*’, and a promoted natural disinterest can be noticed in the rumour ‘*Coronavirus is transmitted via dates*.’ Rumours can have positive intentions behind them, promoting ethics and morality. For example, the following rumour seeks to promote honesty in society: ‘*A Canadian study shows that, every time one lies one loses five hairs from one’s head’*. Here, although the intention or end is ‘good’, the means is ‘bad’.

Rumours are found in this third category to be influenced by certain **attitudes**, be they cultural, political or social (see Table 1). The rumour that ‘*If one stares into the dark, one will be killed by a ghost who thinks you have seen him*’ shows a negative social attitude towards darkness (and indeed ghosts). People seem to have a certain political attitude towards America and its socially perceived political attempt at domination over the world, which can be seen in the following rumour: ‘*WhatsApp conversations and Skype calls are subject to surveillance by America*.’ There appear to be positive social attitudes towards tall individuals, with there being a rumour that ‘*Eating bananas or drinking milk helps one become taller*.’ The feature of having straight hair is socially regarded as a sign of beauty, and for this reason, there is a rumour that ‘*Child’s hair becomes straight when the hair is entirely shaved and then grows again*.’ The far-reaching influence and power of rumours could make companies scared, as any misconduct might cause rumours against their products (Dunbar, 1994; Schoeman, 1994). To illustrate, the following rumour arose from the desire to discipline a company: ‘*It is written on Coca Cola cans, “No God, No Mohammed”’*. On the other hand, companies could themselves utilise rumours to market and promote their own products, for example, initiating rumours such as ‘*Chewing gum helps enhance academic performance*’, thereby promoting the chewing-gum industry. Rumours could also be exploited to cultivate a sense of conspiracy and to manipulate certain international relations. The following rumour illustrates the point: ‘*Some kinds of juices coming from Iran cause bladder cancer’*.

This fourth category demonstrates that rumours can be cultivated via a variety of psychological, social and cultural **values** (see Table 1). One such psychological value is, for example, being mature. The importance of maturity can be seen in the following rumour: ‘*Having white hair at an early age is a sign of reaching maturity at an early age*’. Moreover, psychologically oriented rumours have been used to make people feel good about themselves—for example, to feel good about their ‘flaw’ of having white hair. A social value is given to colours through such rumours as ‘*Black cats are inhabited by ghosts*’. A curse appears to represent a cultural value, around which a number of rumours have circulated: ‘*If one makes fun of the Quran, one may be turned into a sheep*’. Another cultural value is the belief in spirits, which can be seen in the following rumour: ‘*If one cannot get up whatsoever after waking up, this means that a ghost is holding you tightly down*’. In other cultures, many of these sorts of outlandish sayings would be called ‘old wives’ tales’ rather than rumours.

## Discussion

Rumour is not ‘a deviant act’ (Miller, 2006, p. 505). It is rather the natural human response to uncertain situations and the normal social act of addressing uncertainty (Fine and Heath, 2009). It is a cross-cultural phenomenon, being an inevitable ‘congenital’ disorder integral to the constitution of society (Gluckman, 1963). There cannot be society without rumour (Shibutani, 1966). Although rumour is essentially a structural ‘error’ in humanity, the current study has examined the possibility of getting truth out of error (Nietzsche 1996). That is, it has examined whether it is possible to systematically analyse rumours (i.e., *errors*) to see if they can inform us of *truths* about the society wherein they exist. It has empirically shown that, with rumours, one can get some idea about the concerns, interests, attitudes and values of a society. Although one may think that a society should be the one to be looked into to understand its rumours, the current study reverses this logic by looking into rumours in a society to understand the society. That is, this study has checked if rumours in a particular society can inform us of this society. So, the investigation has *not* taken this normal path: society → rumours. Instead, the path for the investigation has been: rumours → society. Traditionally, the conditions that can ease the initiation and diffusion of rumours, innovations and the like are subject to examination, and yet this examination does not look into the rumours and innovations *after* they have been initiated and diffused. In other words, the rumours and innovations should be scrutinised *after* they have been introduced and spread, just as much as they are scrutinised *before* they are introduced and spread. Hence, the current study looks into rumours after they have been introduced and spread.

The current research has found that ‘rumour initiators’ use—whether intentionally or spontaneously (see Knopf, 1975)—certain ‘psychological strategies’ (Prasad, 1935) for convincing others of their own rumours and for enhancing the social acceptance of their claims (cf. McCandless, 2014). Table 2 displays some of these strategies.

**Table 2:** Strategies for enhancing the social acceptance of rumours

Strategy	Description
Appeal to fear	In this strategy, the public is encouraged to believe in a claim by making them feel frightened that certain practices can have dire consequences. These consequences can be, for example, health-related: ‘*The signals of mobile phones can cause cancer’*. These consequences can also be political, such as conspiracies. For instance, ‘*Some kinds of juices coming from Iran cause bladder cancer’*. Other political consequences are control and domination, which can be seen in the following rumour: ‘*WhatsApp conversations and Skype calls are subject to surveillance by America’*
Appeal to the english language	This strategy involves inserting English-language words into claims. Using English-language words (and therefore showing that rumours originate from English-language research) is used perhaps to give rumours credibility, since Saudi Arabs tend to somehow trust English-language research and because English is currently the international language and ‘the language of knowledge’. Besides, many Saudi Arabs do not speak English and thus if the source is shown to be in English, they will not be able to check if the rumours are true and therefore will take it at face value
Appeal to pride	In this strategy, listeners are encouraged to believe in something by supporting their pride in something important to them. This can be social pride. For instance, ‘*William Shakespeare is an Arab’*. Such a claim is expected to be well perceived among Saudi Arabs, given that these people tend to be proud to see the influence of Arab culture on other cultures. Pride can be religious pride. For example, ‘*The two holy mosques have appeared shiny in a picture captured by a satellite*’. Such a claim is expected or hoped (at least by rumour initiators) to be popular among Muslim communities
Appeal to authority	This strategy involves referencing well-respected countries (e.g., America), well-respected ‘leading’ universities (e.g., Cambridge and Florida), journals (e.g., the *British Medical Journal*), media channels (e.g., CNN), companies (e.g., American Wrigley Company) and organisations (Regenstrief Institute). This referencing within rumours is because human society has long experienced a ‘core–periphery’ dichotomy, with a one-way influence from the core to the periphery. The core refers to well-respected ‘senior’ and ‘leading’ organisations and communities, whereas the periphery refers to those outside of this zone of seniority and leadership. The ‘Arab world’ belongs to the periphery and seeks accreditation for its rumours through linking these rumours to the core
Appeal to classification	According to such a strategy, claims tend to be more successful and effective if they seek to classify people. For example, ‘*Intelligent people stay up at night, and stupid people go to bed early*’. In this case, people are classified based on mental abilities, i.e., into clever and stupid people. This classification can be based on gender: ‘*Studies show that men lie three times a day whereas women lie once a day*.’ This classification is, arguably, more likely to be socially accepted if it shows superiority, i.e., if it shows one class to be superior to another, e.g., by women being better than men. Moreover, it appears that judgemental claims are more likely to be socially accepted than non-judgemental claims. By way of illustration, the rumour ‘*Men lie three times a day whereas women lie once a day*’ judges men and women
Appeal to anonymity	This strategy can involve using evidence from unnamed studies or generalised groups (e.g., researchers) to show that a claim is accurate. For example, rumour-initiators may use such vague phrases as: ‘scholars have proven that…’; ‘studies have confirmed that…’; ‘the Western media have widely reported that…’; ‘sources have confirmed that…’; and ‘scholarly articles have confirmed…’, yet without naming these scholars, media channels, sources and scholarly articles. There is no mention of where these scholars, studies and articles come from.⁠⁠⁠⁠ This strategy can also involve making a claim that can never be proven inaccurate because there is no way to check its accuracy. For example, ‘*When one hits walls during the day, ghosts will target one at night*.’ Considering the hidden or ‘transparent’ nature of ghosts and the impossibility of having any communication with ghosts makes it difficult to check any rumour associated with ghosts. Claims are more likely to be successful if they help people predict something that they cannot have ready access to. An appropriate example here is the following claim: ‘*The length of the male organ is related to the length of the nose, foot and finger’*
Appeal to emotion	Claims are more likely to spread if they convey emotions, be they positive or negative. A positive emotion can be luck: ‘*Getting hit by pigeon droppings is a sign of good luck*’. Another positive emotion can involve hope. For example, ‘*Holding wood helps one to achieve one’s wishes’*. An additional positive emotion is healing: ‘*The water that has been used for shisha is good for stomach ache*’. Optimism is another positive feeling: ‘*Having sex in the morning makes one optimistic for the rest of the day’*. A negative emotion is pity: ‘*If a woman drinks too much tea, she suffers from a lack of emotion*’. Another negative feeling is pain or sickness: ‘*When you go from a cold place to a hot place, you will get the flu*’. The appeal can be merely to simple emotion, such as ridicule. For example, ‘*How the female mentality functions is: 70% shopping, 20% how to respond to someone, and 10% love-making. How the male mentality functions is: 70% love-making, 10% how to make love, 10% who to make love with and 10% when to make love*’
Appeal to output	Claims that help with output are common. Such claims may show ways of enhancing productivity or academic performance. For example, ‘*Cows produce more milk when listening to music’* and ‘*Chewing gum helps enhance academic performance*’. These claims may display ways of enhancing wealth: ‘*If a child’s tooth falls out, and then he puts it under his bed, it becomes money when he wakes up’*. On the other hand, these claims can warn against what can prevent productivity, e.g., causing infertility: ‘*Eating lettuce, touching cats, taking vitamin supplements or having the phone next to a man’s organ causes infertility*’

The employment of such strategies suggests that rumour initiators are not, at least politically and socially, naïve and that they do act strategically.

The research has discovered that many rumours are obviously ‘stupid’ (i.e., making limited or no sense, or, at least, being ill-informed, Al Lily et al. 2017), and yet despite this obvious stupidity, individuals still exchange them with each other. This could be seen as a sign of limited critical thinking, education and, moreover, ‘intelligence’. An example of these ‘nonsense’ rumours is ‘*Counting stars causes pimples or tumours*’ – anyone (even those with very little critical-thinking ability) should easily figure out the lack of any correlation whatsoever between ‘counting stars’ and ‘pimples or tumours’. Another example is that ‘*If one lies, one’s nose gets longer*’—again, anyone with even the most limited critical-thinking skills should effortlessly realise how false this rumour is, considering that individuals have lied before and yet their noses have actually remained the same. This rumour appears to come from the children’s tale ‘Pinocchio’, so it was never meant to be taken literally. Another rumour is that ‘*If a child’s tooth falls out, and then he puts it under his bed, it becomes money when he wakes up*’—did anyone not do an experiment on this matter to realise how false this is? Another naïve rumour is that ‘*If one sneezes with one’s eyes open, one’s eyes will come out of one’s face*’—how many times have humans sneezed, while their eyes have remained in place? One may wonder if people actually believe in these rumours, or whether they simply exchange these rumours out of boredom and for entertainment. In their defence, most people automatically close their eyes when they sneeze, making it a hard one to verify.

Interestingly, no ‘political rumours’ were found, perhaps because such rumours are socially seen as ‘off limits’. There were no instructions given to the students to avoid such rumours. Although undergraduates know about politics, they choose not to raise political issues in the context of academia and in Internet searches for rumours.

It has been found that it is common for ‘rumour forwarders’—or, as Buckner (1965) calls them, ‘rumour transmitters’—to sign their rumours with such phrases as ‘*as received’* or ‘*the one to be blamed in case of any possible inaccuracy regarding this piece of information is the one who has sent me this piece*’. These phrases appear to be derived from a widely held belief in Saudi Arabian society that ‘the carrier of bad things is not bad’, implying that the forwarders of ‘bad’ rumours are not ‘bad’. Such phrases have created fertile ground for rumours to be easily and smoothly transmitted throughout society. Such phrases are used so that rumour forwarders can hide behind disclaimers and protect themselves if receivers question or blame them. Rumour forwarders may think that the use of such phrases grants them the freedom to forward ‘anything’ without even reading what they forward. In fact, such phrases actually show rumour forwarders admitting that they have not critically reflected on the received rumours. Besides, thinking themselves protected by such phrases, one person can forward two rumours that show conflicting interests in the same thing. An appropriate example is when one person forwards both: 1) a rumour promoting honesty *(‘If one lies, his nose gets longer’)*; and 2) a rumour promoting dishonesty (‘*Lying a lot makes one live longer’)*. Such phrases have been used by some rumour forwarders to show a sense of ethics, referencing and honesty in communicating and passing around information. They may also suggest that some rumour forwarders do not necessarily believe in (and/or, more importantly, understand) the content of the forwarded messages.

## Conclusion

This article has studied the perceptions of male undergraduates’ views of a broader trans-national Arabic-writing audience on the Internet. It has underpinned the theoretical proposition that ‘rumours often serve as a window into a community’ (Kelley, 2004: v). It has suggested that scholarly rumours (perhaps like non-scholarly rumours) mirror the public temper of a society, reflecting a range of emotions from simple to complex, from concerns, interests and attitudes to values. Simpler emotions (e.g., concerns) are, perhaps, more easily affected by scholarly rumours than more complex emotions (e.g., values). A society produces scholarly rumours based on its public temper, and therefore these rumours are a deep, complex reflection of its public temper. Hence, scholarly rumours can be effective ‘informants’ of public tempers. Scholarly rumours capture the specific and changing emotional aspects of the milieu in which these rumours exist. They reveal why habitants are committed to these rumours. They form ‘books’ in which the emotional formula of a society is written and may be transmitted to succeeding generations. They can exist as ‘inscribed spaces’ on which a certain society ‘writes’ their temper. They can serve the function of public tempers, even though individuals can be drawn to rumours without being conscious of any desire to promote a public temper (Baumeister et al. 2004). Although scholarly rumours are ‘dark spots’, this study has examined whether it is feasible to get ‘light’ out of this ‘darkness’, i.e., to use scholarly rumours (i.e., dark spots) as indirect informants of truth (i.e., light) about society, meaning that something can come out of its opposite. It is not only that components of a given culture can be predicted from its rumours, but also that rumour (just like language) is a function of a given culture. Lakoff (2010) contends that linguistic metaphors—just like rumours—play an essential role in the functionality of human socio-political life and in the direction of societies (particularly their politics).

This article helps give a sense of what is perceived as rumour in a social context within the Arabic-speaking world. It provides the English-speaking world with an example of how an Arab society ‘thinks’. This is, however, not meant to reduce the various Arab cultures into one single culture, to create essentialism, to (re)produce a form of orientalism or to promote a neo-colonial style overgeneralisation about the ‘Arab mentality’. The findings of the study are, at their best, applicable merely to the *Saudi* Arabian mentalities, which may, or may not, share the same characteristics as other Arabian mentalities. It should be remarked that this article is not about ‘Arabic rumours’ since rumours cannot be ethnicity-specific (e.g., white people’s rumours). It, instead, is concerned with how a certain group (here, Saudi Arabs) deals with rumours in their own language (here, Arabic) or translates rumours into their language. The current study is purely qualitative, and therefore this area would benefit from further research that quantifies its findings. The current research has shown that the public temper of a society can be understood through the nature of its rumours, and therefore further research should be carried out to reverse this investigation, by examining whether one, through understanding the public temper of a society, can design a model that predicts the kinds of rumours that exist and will exist in this society. That is, further research should examine whether the nature of rumours can be gleaned through understanding the public temper of a society. Further research should look into how certain societies, organisations or individuals may influence and manipulate the public temper of other societies, organisations or individuals by means of the political power of rumours.

Further research should also consider a political process wherein rumours are influenced by themselves. This influence may take two forms. First, the enactment of one rumour may hold back or conflict with existing rumours, or disrupt the possibility of enacting other rumours. Second, rumours may feed on themselves, making more rumours possible. To illustrate, a rumour may require another new rumour in order for the two to function effectively. New rumours may obligate their forwarders to link them to other rumours, establishing an almost independent network of political power. Rumours may appear to have a power and a life of their own, with the role of humans becoming less relevant. While rumours do not make themselves, and are constituted by humans, humans may not afterwards have full freedom to determine how rumours grow and shape the public temper. In this case, rumours get out of hand and shape themselves, away from the agency of humans. Indeed, it is not only researchers but humans in general that hardly recognise the implicit ability of non-humans (here, rumours) to play politics and act as politicising media.

Further studies should be carried out to re-apply the current research to Saudi women’s universities, older people and younger people (e.g., high-school students). Transnational comparisons within the Arab world would be also worthy of academic investigation using the methodology of the current research. Likewise, comparative studies in how rumours are evaluated in the public tempers of other Muslim areas can be of considerable value, especially considering that the Koran warns against rumour-forwarders: ‘O you who believe! If a Fāsiq (liar–evil person) comes to you with any news, verify it, lest you should harm people in ignorance, and afterwards you become regretful for what you have done’ (The Dwellings Chapter, Verse 6).

### Data availability

All data analysed or generated are presented in the paper.
